# Metabolic guidance and stress in tumors modulate antigen-presenting cells

**DOI:** 10.1038/s41389-022-00438-y

**Published:** 2022-10-16

**Authors:** Jaeoh Park, Limei Wang, Ping-Chih Ho

**Affiliations:** 1 Department of Fundamental Oncology, University of Lausanne, Lausanne, Switzerland; 2 Ludwig Institute for Cancer Research, University of Lausanne, Epalinges, Switzerland

**Keywords:** Immunology, Cell biology

## Abstract

Successful antitumor immunity largely relies on efficient T cell priming by antigen-presenting cells (APCs); however, the capacity of APCs is found to be defective in many cancers. Metabolically reprogrammed cancer cells support the energetic and biosynthetic demands of their high proliferation rates by exploiting nutrients available in the tumor microenvironment (TME), which in turn limits proper metabolic reprogramming of APCs during recruitment, differentiation, activation and antigen presentation. Furthermore, some metabolites generated by the TME are unfavorable to antitumor immunity. This review summarizes recent studies on the metabolic features of APCs and their functionality in the TME. Particularly, we will describe how APCs respond to altered TME and how metabolic byproducts from cancer and immunomodulatory cells affect APCs. Finally, we introduce the current status of APC-oriented research and clinical trials targeting metabolic features to boost efficient immunotherapy.

## Introduction

Tumor is akin to a chronic infection where immune cells are constantly exposed to an antigen that cannot be cleared. Upon the encounter of antigens, the innate immune cells, such as dendritic cells (DCs) and macrophages, play a role as the first-line of defense against antigens by uptaking in an indiscreet manner. DCs, macrophages, and B cells are usually referred to as professional antigen-presenting cells (APCs) and are critical “matchmakers” which awaken antigen-specific adaptive immunity via presenting peptides loaded on major histocompatibility complex I or II (MHCI/MHCII) to T lymphocytes in the lymph nodes (LNs). Cells referred to as “non-professional APCs” by expressing MHCII in an inducible manner, for example, innate lymphoid cells, fibroblast, or epithelial cells, also participate in the activation of adaptive immunity [1].

The ability of T cells to kill tumor cells in an antigen-specific manner is conferred by APCs as so in infection, therefore, the role of APCs is critical for the successful immunosurveillance of cancer. Although clonal expansion and differentiation of T cells majorly occur within lymphoid organs, a positive correlation between tertiary lymphoid structures (TLS) for priming intratumoral T cells with better response to therapy or prognosis implies the importance of APCs within TME [2–4]. However, immunosurveillance is not always successful because tumor cells render TME felicitous to themselves by depleting nutrients and secreting metabolites.

Immune cells co-habitating with tumor cells compete for nutrients with limited availability, and are constantly communicating with tumor cells within the TME. Competition for nutrients and exposure to TME-derived metabolites lead to the metabolic adaptation of immune cells, and consequently result in dysfunctionality in antitumor immunity. DCs frequently show disrupted function, and tumor-associated macrophages (TAM) rather play pro-tumorigenic roles within the TME. Therefore, targeting metabolic crosstalk in the TME is rising as a novel therapeutic strategy. In this review, we will address the immunometabolic features of APCs, particularly focused on DCs and TAMs, and their crosstalk within the TME. Additionally, we will also discuss the studies of adaptation and functional alteration of APCs by tumor metabolites and the impact on antitumor immunity. We will further discuss the therapeutic approaches targeting the metabolic features of APCs to restore their antitumor immunity.

## Cell biology of APCs in cancer

### Macrophages

Although TAMs adopt more diverse phenotypes than dichotomized status as their heterogeneity within TME has been actively discussed in recent studies, the activation status of macrophages has long been oversimplified into two categories: classically activated, pro-inflammatory M1 macrophages and alternatively activated, anti-inflammatory M2 macrophages [3–5]. M1 macrophages are believed to be anti-tumorigenic by secreting pro-inflammatory cytokines such as tumor-necrosis factor alpha (TNF-α), interleukin (IL)-1β, and IL-6, while M2 macrophages are known to be pro-tumorigenic due to their expression of anti-inflammatory molecules such as IL-4, IL-10, and transforming growth factor beta (TGFβ). M1 macrophages and M2 macrophages adopt different metabolic features. Dependency on glycolysis is a crucial feature for M1 macrophages, since perturbation of glycolysis alters their polarization status into M2 macrophages [6, 7]. The pentose phosphate pathway (PPP) enhanced in M1 macrophages provides nicotinamide adenine dinucleotide phosphate (NADPH) for the production of nitric oxide (NO) and reactive oxygen species (ROS) [8]. In contrast, M2 macrophages adopt oxidative phosphorylation fueled by fatty acid oxidation (FAO) and glutamine metabolism. Due to unfavorable conditions to support the M1 polarization of macrophages, TAMs are often featured as M2 macrophages. How TME alter the polarization status of macrophages will be discussed further in the following sections.

The abundance of TAMs has been associated with poor prognosis in different types of solid tumors, since TAMs carry out a pro-tumorigenic role via triggering T-cell dysfunction [9]. B7 superfamily 1 (B7S1) and CD39 expressed on TAMs have been shown to be involved in the direct suppression of T cell activity [10, 11]. Cytokines that TAMs secrete are involved in inhibiting T-cell activity as well. For example, IL-10 secreted by TAMs enhances *N*-glycan branching, which in turn curtails the sensitivity of T cells to the antigen [12]. Moreover, TAMs recruit CCR6-positive regulatory T cells (Treg) to render the TME into an immunosuppressive milieu in colorectal cancer models, and the infiltration of Tregs driven by TAMs has further been studied in ovarian cancer and laryngeal squamous cell carcinoma [13–15]. As the role of TAMs in TME is appreciated to be suppressing antitumor immunity, inhibition of TAM infiltration into TME via colony-stimulating factor 1 receptor (CSF1R) blockade treatment resulted in a significant reduction of tumor growth accompanying increase of T cell infiltration [16].

Since efficient activation of T cells is largely dependent on APCs, antigen-presenting capacities of APCs within TME has long been under extensive investigation. However, the roles of TAMs as APC within TME have been less appreciated compared to those of DCs. TAMs within TME adopt an M2-like phenotype, which express low level of MHCII, thereby exhibiting limited antigen-presenting capacity. Furthermore, low expression of CCR7, critical for LN migration, is another factor that limits their ability as APC, rendering macrophages less effective compared to DCs. A study using ascites-derived DCs or macrophages from human ovarian cancer patients showed a superior capacity of DCs activating CD8 T cells [17]. Inhibition of signal regulatory protein α (SIRPα) with CD47 blockade efficiently enabled DC-mediated cross-priming of CD8 T cells, whereas macrophages were incapable to do so [18]. In contrary to these reports, CD169^+^ macrophages in the LN have been shown to play critical roles in exerting antitumor immunity by cross-presenting dead cell-associated antigens to CD8 T cells [19]. Further studies using in vitro models demonstrated that macrophages can cross-present antigens to prime CD8 T cell response [20, 21]. Moreover, a recent study revealed the capability of CD206^+^ macrophage subset to cross-present tumor antigens [22]. Therefore, further studies on the unique potential of TAMs in priming T cells within the TME are needed, which will not only expand our understanding on their unappreciated functional diversities but also potentiate therapies targeting APCs to enhance antitumor immunity.

### Dendritic cells

DC is another quintessential APCs of the immune system, especially for the activation of antitumor T cells [23–26]. There are three types of DCs found in the TME, and can be distinguished as plasmacytoid DCs (pDCs), conventional DCs (cDC1 and cDC2), and monocyte-derived DCs (moDCs) based on their phenotypical and functional properties [27]. Owing to their responsibility for the initiation of the “cancer-immunity cycle”, cDC1 (CD8α^+^CD103^+^BATF3^+^ CLEC9A^+^XCR1^+^) is identified as a critical APC subset for tumor antigen drainage and robust T cell activation [28, 29]. cDC1s generally appear to be more dependent on OXPHOS, displaying higher mitochondrial mass and ΔΨm than cDC2s [30]. Upon immunogenic activation, cDC1s elevate glycolysis and lactic fermentation [31]. Indeed, interruption of glycolysis impairs not only the maturation, but also the immunogenicity and T cell stimulatory capacity of DCs [30]. In line with their dependency on glycolysis, glucose deprivation impairs DC-mediated immune response by affecting the motility of splenic CD11c^+^ cDCs and oligomerization of CCR7, whose level correlates with T cell infiltration and patient survival [28, 32]. However, mitochondrial respiration appears to be as important as glycolysis induction for proper cDC1 activation. Splenic cDC1s in aged mice that have mitochondrial dysfunction (decrease in basal OCR and increase in proton leakage and ROS) displayed reduced endocytic activity and antigen presentation capacity [33]. In addition, bone marrow-derived cDC1-like cells have been shown to upregulate de novo fatty acid synthesis (FAS) to accumulate phospholipids upon stimulation [34]. As cDC1s are a crucial subset for effective antitumor immunity, clarifying how these metabolic pathways are intertwined in regulating the activity of cDC1s within TME will be important.

cDC2, another subset of conventional DCs, can be identified by high expression of CD11b, CD1c (human), and SIRPa (CD172a). In contrast to cDC1, ROS strongly skews the differentiation toward cDC2. Likewise, in cDC1s, upregulation of glycolysis and FAS are required for the activation and maturation of cDC2s [35]. This reflects the reason why DCs with high lipid content are more potent in priming T cells [36]. In addition to the essential roles of cDC1s in the induction and maintenance of antitumor immunity, cDC2s can effectively elicit intratumoral CD4 T cell responses and induce the polarization of diverse subsets of CD4 Th cells [37, 38]. Upon Treg depletion, cDC2 can migrate to the draining LN and improve CD4 T cell differentiation in vivo. Similarly, a high level of cDC2s is strongly associated with longer progression-free survival and higher infiltration of CD4 T cells in both melanoma and human head and neck squamous cell carcinoma (HNSCC) [38].

Although pDCs are mainly known for antiviral immunity, they are a potential APC subset in TME as well [39, 40]. pDCs are derived from either myeloid common DC progenitor (CDP) or IL-7R^+^ lymphoid progenitor cells, but single-cell RNA-seq (scRNA-seq) demonstrated that only myeloid-derived pDCs show similar potential to process and present antigens as cDCs [41]. Reduced expression of co-stimulatory molecules and type I interferon (IFN) upon 2-deoxyglucose (2-DG) treatment demonstrated the importance of glycolysis during activation and maturation of pDCs. However, the analysis of the whole transcriptome of human pDC upon toll-like receptor (TLR) 7/8 stimulation proved the induction of glutaminolysis and OXPHOS [35]. Further studies are needed to demonstrate the metabolic dependency of pDC differentiation. The role of pDCs in anti-cancer immunity remains controversial [41]. Although pDC infiltration is associated with poor outcomes in human breast cancer, pDC can induce tumor regression through type I IFN-mediated mechanism following intratumoral injection of TLR7 ligand in orthotopic murine mammary tumor model [39].

In addition to the DC subsets listed above, different types of DCs from human peripheral blood and tumors as well as from murine tumor models have been recently identified through scRNA-seq. For instance, AXL^+^SIGLEC6^+^ cells (AS DCs), a new subdivision between cDC-like and pDC-like cells have been demonstrated to potently activate T cells [42]. cDC2A and cDC2B, two principal cDC2 lineages, identified by combining RNA-seq and chromatin accessibility analyses with genetic reporter expression, have distinct pro- and anti-inflammatory potential and are characterized by distinct metabolic states [43]. Mature DCs enriched in immunoregulatory molecules (mregDCs), co-expressing immunoregulatory genes (*Cd274*, *Pdcd1lg2*, and *Cd200*) and maturation genes (*Cd40*, *Ccr7*, and *Il12b*), can uptake tumor antigens, upregulate IL-12 in an IFNγ-dependent manner, and initiate effector T cells response [44]. DC3s, identified as the CD88^−^CD1c^+^CD163^+^ subset, share similar secretory profiles of immune modulators with monocytes and cDCs. Of note, infiltration of DC3s positively correlates with the expansion and abundance of resident memory T cells in human breast cancers [45].

### B cells

B cells are another type of APCs that participate in antitumor immunity by activating T cells via MHC-loaded tumor antigens and by secreting tumor-reactive antibodies. Within TME, B cells are frequently found in TLS, where multiple types of immune cells cluster to form a structure resembling secondary lymphoid organs. Mature TLS contain an organized B cell zone surrounded by T cells exerting a humoral response of antitumor immunity. Therefore, the presence of TLS has been correlated with a better prognosis of tumor patients and better response to immunotherapies in different types of cancers [46, 47]. Furthermore, patients with colocalization of CD20^+^ B cells with CD8 T cells showed longer survival than the patients with CD8 T cells alone in ovarian cancer [48]. In contrast, infiltration of B cells into TME has been reported to promote the progression of tumors as well. Together with earlier studies showing a better response to therapy upon B cell depletion, the population called “regulatory B cell (Breg)” secreting immunosuppressive cytokines (IL-10 and IL-35) is appreciated to be responsible for pro-tumorigenic activity of B cells [49, 50]. Nevertheless, as a rising number of studies indicate a positive prognostic value of B cells, the distinct roles of heterogenic B cell subsets should be precisely evaluated for a better therapeutic application.

## Metabolism of APCs in TME

Tumors can manipulate TME to support their proliferation, and promote metastatic behavior and therapeutic resistance, in part through inducing APC tolerization by enriching TME with immunosuppressive factors that can suppress anti-tumoral activities of APCs.

One common feature of most solid tumors is dysfunctional vascularization which causes severe hypoxia [51]. Hypoxia induces dual consequences on the migration of DCs based on the duration [52, 53]. Short-time hypoxia results in a better migration of moDCs, while long-term hypoxia inhibits the migration of DCs [52]. The hypoxia-inducible factor 1 (HIF1α) expression is inhibited by direct binding of long non-coding RNA Dpf3 (lncRNA-Dpf3), and this further impairs glycolysis and inhibits CCR7-mediated DC migration [53]. Availability of oxygen affects the feature of TAMs as well. The expression of regulated in development and DNA damage response 1 (REDD1) is upregulated in TAMs under hypoxia to support angiogenesis and pro-tumoral phenotype, and fine-tunes the M2 phenotype of TAMs [54, 55]. Association between hypoxia and M2 TAMs is further appreciated by their distribution within the TME. TAMs adopting M2-like feature distribute in the hypoxic region, while M1 TAMs infiltrate into the normoxic region [56]. Although the cue for this distribution seems not yet clear, TIE2 expression on TAMs has been shown to play a role in the guidance to the vasculatures [57]. TAMs with stabilized HIF1α under hypoxia can induce the expression of vascular endothelial growth factor (VEGF) and the differentiation into M2 TAMs [58]. Furthermore, TAMs further enhance the hypoxia of tumors forming a feed-forwarding loop to modulate tumor metabolism, which subsequently results in therapeutic inefficiency [59]. Hypoxia might have adverse effects on proper B cell activities within TME as well. Forced stabilization of HIF1α by depleting Von Hippel-Landau tumor suppressor protein (pVHL) in B cells resulted in the reduction of clonal expansion, recall response, and high-affinity antibody production via perturbation of mTORC1 activity [60]. Furthermore, stabilization of HIF1α has been shown to drive IL-10 expression in B cells in encephalomyelitis [61]. Although not many studies are done on metabolic aspects of B cells within TME, these studies show the potential involvement of hypoxia in hampering antitumor immunity exerted by B cells via limiting humoral response and driving IL-10 secreting regulatory B cell development.

Fatty acid (FA) enriched in the TME has been shown to contribute pro-tumorigenic phenotypes of TAMs. CD36 has been demonstrated as the receptor responsible for the uptake of lipids in TAMs, and these TAMs utilized FAO as an energy source. Enhanced FAO facilitates the generation of pro-tumoral TAMs via JAK1-STAT6 signaling, which is activated by a high level of oxidative stress due to increased ROS production [62]. Recently, tumor-derived glucosylceramide has been demonstrated to induce M2 polarization of macrophages via triggering ER stress [63]. Furthermore, TAMs accumulated with lipids via caspase-1 activation have been shown to exhibit pro-tumoral properties [64]. The lipid metabolism of TAMs has further been featured in several studies. M2 TAMs in ovarian cancer have been shown to adopt deregulated peroxisome proliferator-activated receptor (PPAR) signaling due to the accumulation of oxidized low-density lipoprotein (LDL) in the TME [62]. Perturbation of lipid uptake sufficiently ameliorated pro-tumorigenic potential of TAMs, showing a critical role of lipid metabolism in pro-tumoral TAMs [65]. Prominent use of lipid metabolism by M2 macrophages also reflects their survival advantage over M1 macrophages within lipid-rich TME. Glutamine metabolism is another way that TAMs depend on to produce energy. Depletion of glutamine synthetase (GS) polarize TAMs into M1-like TAMs, which consequently led to the inhibition of metastasis [66]. Furthermore, M2 skewing of macrophages by high level of α-ketoglutarate (αKG) via glutaminolysis suggested therapeutic potential of targeting glutamine metabolism of macrophages within TME [67].

The role of lipid, particularly triglycerides (TG), in DCs has also been reported [68]. Upregulation of scavenger receptor A expressed on DCs leads to the uptake of lipids from TME [68, 69]. In addition to the uptake of lipids, alteration of metabolic pathway can promote the synthesis and accumulation of FAs within DCs. Dysfunctional DCs prefer to use FAs as the carbon source via augmenting FAO instead of glycolysis [70]. In ovarian cancer, DCs exhibit ER stress response and drive IRE1a/XBP1 activation, leading to the synthesis and accumulation of FAs and TG [71]. In melanoma, tumor cells activate WNT5a/β-catenin-PPARγ signaling, which in turn upregulates the expression of carnitine palmitoyltransferase-1a (CPT1A) to promote FAO [72]. FA-carrying tumor-derived exosomes (TDEs) drive DCs to activate PPARα signaling pathway and promote FAO by increasing intracellular lipids [73]. Several studies have shown that abnormal accumulation of lipids in DCs is one of the major factors impairing antigen cross-presentation [74]. Oxidized lipids, especially electrophilic oxidatively truncated lipids (ox-tr-LB), which covalently adduct with heat shock protein 70 (Hsp70), mainly cause defective trafficking of peptide-MHCI (pMHCI) complexes from phagosome/lysosome to the cell surface [75]. Furthermore, lipid accumulation in DCs can downregulate CD86 and upregulate tolerogenic cytokine IL-10 [76]. These studies demonstrate the importance of lipid metabolism regulating the functionality of DCs within TME.

## Metabolites that can act as immune signaling molecules in APCs

Metabolites produced by tumors such as lactate, succinate, and αKG are utilized not only for feeding metabolic pathways, but also function as signaling molecules to the neighboring cells (Table 1). The immunoregulatory function of these metabolites are accentuated as a modulator of TME, which are now drawing attention as therapeutic targets to awake antitumor immunity.

**Table 1:** Metabolites and their effects on APC function.

TME metabolic products/metabolites	Effect on APCs and immune consequences	Mechanism of APC tolerance	Reference
Low pH	Favor moDC differentiation; Obstruct antigen uptake and destabilize antigen-MHCI complex	Increase mitochondrial respiration; Inhibit mTORC1 activity; DEC205 conformational change	[77, 83, 84]
WNT5a	Decreased CD103^+^DCs infiltration; IDO1 production; Treg generation	β-catenin activation	[72, 136]
	Promote FAO process	PPARγ upregulates the expression of CPT1A	[72]
Prostaglandin E2	Production of IL-6, CXCL1 and G-CSF; Type I IFN elimination	?	[137]
Hypoxia	Short-time: moDCs have a better migration; Long-time: IDO and adenosine (DCs) VEGF and differentiation into M2 TAMs	Long-time - HIF1α expressing; lncRNA-Dpf3	[52, 58]
	Angiogenesis and fine-tunes M2 phenotype	Upregulate REDD1	[54, 55]
	Vasculatures	ANG2/TIE2 axis	[57]
Glucosylceramide	M2 polarization	ER stress	[63]
Lipid	Pro-tumoral properties	Caspase-1	[64]
	Utilize for FAO	PPAR and CD36 expressing	[62, 65]
	Downregulate CD86 and upregulate IL-10	?	[76]
ox-tr-LB	Reduced antigen processing ability; Impair CD8 T cell response	ox-tr-LB covalently adduct with Hsp70	[75]
Fatty acid-carrying TDEs	DCs intracellular lipid content and mitochondrial respiration	Upregulate PPARα signaling pathway, promotes FAO, and enriches lipid droplets	
TME	Lipid body accumulation; Antigen presentation	ROS/4-HNE adducts/ER stress/XBP1	[71, 73]
Lactate	M2 polarization	Upregulate M2-associated genes (e.g., Arg1 and VegfA); Olfr78/GPCR/ICER; Epigenetic modulator	[58, 78, 80, 81]
Lactate	Decrease MHCII, cAMP, IL-6, and IL-12	GPR81 expression (DC)	[82]
Succinate	Migration of TAMs into TME	PI3K/HIF1α cascade	[85]
Glutaminolysis	Alternative activation of macrophages	Jumonji-domain-containing protein-3	[67]
2-HG	M2 polarization; T cell dysfunction via CD39 expression	Kynurenine/AhR/NF-κB/KLF4 cascade	[11, 88]

Overview of TME metabolites reprogram APCs to elicit APC-mediated anti- or pro-cancer immunity.

*IDO1* indoleamine 2,3-dioxygenase, *FAO* fatty acid oxidation, *PPARγ* peroxisome proliferator-activated receptor-γ, *CPT1A* carnitine palmitoyltransferase-1a, *HIF1α* hypoxia-inducible factor 1α, *VEGF* vascular endothelial growth factor, *REDD1* regulated in development and DNA damage response 1, *ANG2* angiopoietin 2, *ER* endoplasmic reticulum, *Ox-tr-LB* oxidatively truncated lipids, *TDE* tumor-derived exosomes, *2-HG* 2-hydroxyglutarate, *AhR* aryl hydrocarbon receptor, *KLF4* kruppel-like factor 4, ? unknown.

**Fig. 1:**
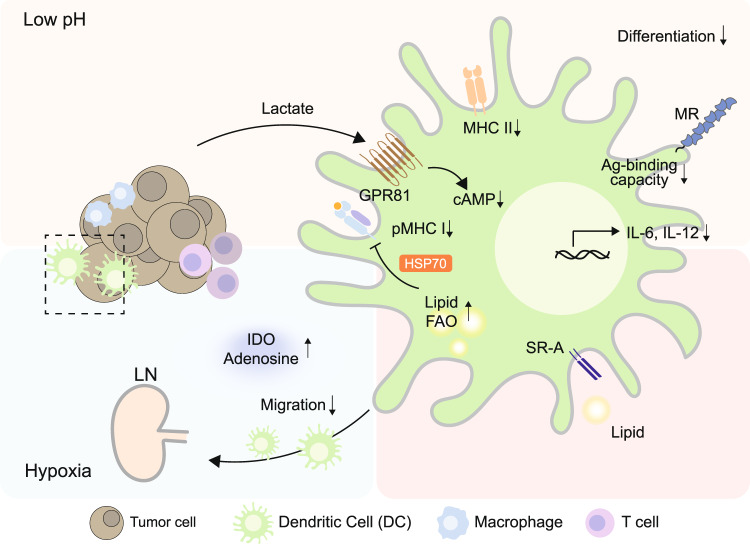
Metabolic alteration of TME affecting the immunogenic function of DCs. Low pH resulting from lactate from tumor cells leads to the downregulation of cAMP, IL-6, and IL-12 by stimulating GPR81. Furthermore, lactate limits the APC function of DCs by suppressing MHCII-mediated antigen presentation and inducing a conformational change of mannose receptor (MR), which is responsible for antigen (Ag) binding. A hypoxic environment limits the migration of DCs to the LN, and enrichment of IDO and adenosine induce DCs to express immunosuppressive cytokines. Fatty acid oxidation (FAO) fueled by lipids in TME is prominently used by dysfunctional DCs that are impaired with antigen cross-presentation. Lipid uptake by scavenger receptor A (SR-A) results in the accumulation of lipids within DC, which consequently affects peptide-MHCI (pMHCI) trafficking by forming an adduct with heat shock protein 70 (HSP70).

Low extracellular pH reduces glucose consumption and lactate production, increases mitochondrial respiration, and inhibits mTORC1 activity. These environmental factors govern the differentiation of monocytes into DCs [77]. Furthermore, lactate produced by highly glycolytic tumor cells are considered an immunomodulatory molecule [78], inducing metabolic polarization of macrophages into M2 TAMs by triggering the expression of M2-associated genes (e.g., arginase 1 (*Arg1*) and *VegfA*) in a HIF1α-dependent manner [58]. An investigation of receptors responsible for lactate sensing demonstrated that the odorant receptor OLFR78, a type of G protein-coupled receptor (GPCR), as a responsible sensor for lactate uptake by TAMs in TME [79]. GPCR-mediated inducible cyclic AMP early repressor (ICER) has further been shown to dictate lactate as a pro-tumorigenic signal altering TAMs to obtain M2-like features [80]. Macrophages seem to engage lactate as an epigenetic modulator as well. Although histone lactylation has only been demonstrated in a bacterial infection model, preferential lactylation in macrophages in the resolving phase rather than the pro-inflammatory phase suggests possible roles of lactate in sculpting pro-tumorigenic function of TAMs [81]. Lactate can also limit antigen-presenting capacity of DCs by stimulating G protein-coupled receptor 81 (GPR81) expression, which downregulates cell surface expression of MHCII and decreases cAMP, IL-6, and IL-12 [82]. Acidification of TME by lactate limits antigen uptake and destabilizes the antigen-MHCI complex [83]. Furthermore, low pH reduces the antigen binding capacity of mannose receptor (MR), such as DEC205, expressed on a variety of APCs, including DCs, by inducing conformational change (Fig. 1) [83, 84].

**Fig. 2:**
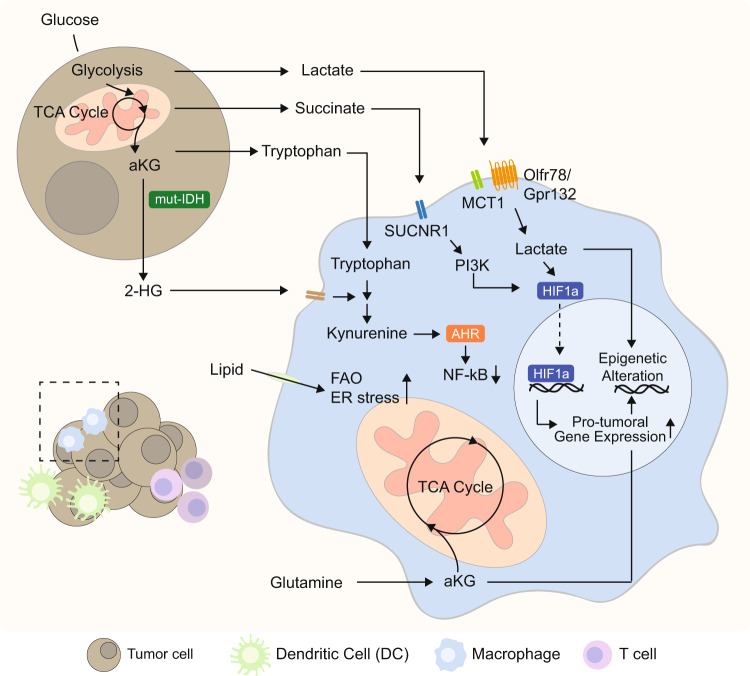
Metabolic interplay between tumor cells and TAMs within TME. Lactate transported by MCT1 and GPCRs (e.g., OLFR78 and GPR132) shapes pro-tumoral properties of TAMs by upregulating M2-associated genes. Enrichment of TCA cycle metabolites affects the metabolic properties of TAMs. SUCNR1-mediated succinate uptake activates PI3K-HIF1α signaling to induce polarization of TAMs. Furthermore, accumulation of kynurenine within TAMs generated from tryptophan and 2-HG (generated by mutant IDH on tumor cells) by sequential activities of IDO and TDO, binds to aryl hydrocarbon receptor (AhR), and suppresses NF-κB signaling. Glutaminolysis produce α-ketoglutarate (αKG), supplying intermediate for the TCA cycle, and further alters the epigenome of macrophages. Increased dependency on fatty acid oxidation (FAO) or ER stress induced by lipids enriched in TME leads macrophages to adopt pro-tumoral characteristics.

Metabolites from the tricarboxylic acid (TCA) cycle not only serve as metabolic precursors, but also actively play a role as messengers between tumor cells and immune cells. Tumors harboring mutations on TCA cycle enzymes, such as succinate dehydrogenase (SDH), fumarate hydratase (FH), and isocitrate dehydrogenase (IDH), reshape TME, which in turn affect immunosurveillance. Succinate, as a ligand of succinate receptor 1 (SUCNR1), conveys signal to phosphoinositide 3-kinase (PI3K)-HIF1α cascade within TAMs and promotes the migration of TAMs into TME [85]. 2-hydroxyglutarate (2-HG), an analogous metabolite produced by mutant IDH, has been suggested to lead dysfunctionality of TAMs via altering tryptophan metabolism [86]. Alteration of tryptophan metabolism is one of the strategies that tumor utilizes to suppress antitumor immunity. IDO, a rate-limiting enzyme of tryptophan catabolism, is found upregulated in a wide range of cells, including tumor cells and immune subsets. Reduced availability of tryptophan due to high levels of IDO within TME induces T cell dysfunction within TME. Likewise, in tumors with mutant IDH, attenuation of Bar-adapter encoding gene 1 (BIN1) expression in human cancers has also been demonstrated to alter tryptophan metabolism by promoting IDO activity [87]. Pro-tumoral macrophage is another compartment that express a high level of IDO. IDO that is highly expressed in TAMs further contribute to perturb antitumor immunity by depleting tryptophan but enriching metabolic product, kynurenine (KYN), which functions as the ligand for aryl hydrocarbon receptor (AhR). KYN suppresses the nuclear factor kappa light chain of activated B cells (NF-κB) pathway involving kruppel-like factor 4 (KLF4), and leads to M2 polarization and T cell dysfunction via CD39 expression [11, 88]. As the role of KYN has been implicated in the formation of immunosuppressive TME, its depletion via the administration of kynureninase reversed immunosuppressive TME. However, whether this involves the modulation of the activity of TAMs is not clear (Fig. 2) [89]. Accumulation of IDO will also render DCs to further contribute to the alteration of TME into immunosuppressive TME [90]. Depletion of tryptophan within TME markedly decreases Ag uptake and the expression of CD40 and CD80 on DCs. In addition, DCs under tryptophan-deprived conditions significantly increase inhibitory receptors (ILT3 and ILT4), which leads to the induction of Tregs [91]. KYN has been evidenced to be strongly correlated with cDC2 differentiation. IDO1-expressing cDC1s induce regulatory cDC2 through the secretion of L-KYN. This, in turn, recruits the AhR-activated cDC2 subset into a tolerogenic pool. Since the importance of KLF4 in regulating functionality and fate specification of cDC2s have been reported, whether tolerance of cDC2 induced by KYN within TME involves KLF4 should be further investigated [92]. Furthermore, IL-12 plus GM-CSF-treated tumor-bearing mice induce long-term maintenance of IDO^+^ DC in the tumor-draining LNs (TDLNs) through transient induction of IFNγ. In vitro system modeling demonstrated that IDO expression is maintained by a positive feedback loop between IDO-KYN and AhR-IDO. This renders DCs to be tolerogenic, inhibiting T-cell proliferation as well as inducing Treg differentiation [93]. Tregs generated in the TDLN can further upregulate IDO expression in DCs via CTLA4/B7 interaction [94]. In addition to IDO, adenosine accumulated within hypoxic TME stimulates A_2B_ adenosine receptor, skewing DC differentiation into a distinct subset that secretes high levels of immune suppressors, pro-inflammatory and tolerogenic factors such as IL-6, IL-8, IL-10, and TGFβ [95]. Taken together, TME enriched with metabolites secreted by tumor cells actively modulate metabolic phenotypes and functionalities of APCs into pro-tumorigenic APCs.

## The impact of tumor aggressiveness on the function of APCs

The dysfunction of APCs is largely driven by the metabolic rewiring of tumor cells. However, whether the metabolic features of APCs are altered gradually depending on the progression of tumor or how the tumor aggressiveness and properties affect APCs is unclear. Below, we introduce studies regarding how APCs are affected by two indicators of tumor aggressiveness; cancer stem cell (CSC) and epithelial-to-mesenchymal transition (EMT).

CSC has been described to be responsible for treatment resistance, tumor metastasis, and tumor recurrence. Of note, the maintenance of CSC highly relies on the ADP-ribosylation factor 1 (Arf1)-mediated lipolysis pathway that provides energy from FA. Disruption of lipid metabolism by Arf1 ablation enhanced antitumor immunity exerted by DC via triggering ER stress and secretion of damage-associated molecular patterns (DAMPs) [96]. This indirectly shows how CSC can escape antitumor immunity. Furthermore, accumulation of ROS, which correlates with tumor aggressiveness, due to oxidative stress or hypoxia within TME leads to the upregulation of heme oxygenase 1 (HO-1) expression in DCs and TAMs [97, 98]. The induction of HO-1 in APCs profoundly contributes to the shaping of immunosuppressive TME. HO-1 expression in DCs inhibits lipopolysaccharide (LPS)-induced phenotypic maturation and production of pro-inflammatory cytokines, which hinders T-cell proliferation, and increases IL-10 production [99]. Moreover, an enhanced beneficial effect of antitumor vaccine by ablation of HO-1 in TAMs demonstrates how the abundance of ROS and aggressiveness of tumor can affect the function of APCs [100].

EMT is another feature that is associated with tumor aggressiveness in many cancers. Compared to differentiated tumors that still retain epithelial features, tumor cells undergoing mesenchymal transition gradually rewire their metabolism to support their energy demand. TGFβ, a well-known inducer of EMT, drives the upregulation of glycolysis via inducing the expression of glycolytic enzymes in glioblastoma cells [101]. Additionally, transcription factors (*Snail*, *Slug*, *Twist*, and *Zeb1*) involved in the process of EMT commonly upregulate glycolysis, while they downregulate mitochondrial respiration [102–105]. For example, SNAIL enhanced glycolytic flux by increasing glucose uptake and downregulating the expression of fructose-bisphosphatase 1 (FBP1) via suppressing promoter activity [104]. In a murine pancreatic cancer model, the tumor deficient of *Zeb1* lacked a glycolytic switch upon the blockage of OXPHOS with oligomycin treatment [106]. An increase of glycolysis in tumors undergoing EMT will shape the TME into a lactate-rich environment, which will further perturb proper antitumor immunity. In line with this, *Zeb1* induction in breast cancer has been demonstrated to be critical for pro-tumoral macrophage polarization [107]. This intrigues the question of whether the ablation of lactate signaling in TAMs will alleviate the aggressiveness of tumor. Furthermore, The Cancer Genome Atlas (TCGA) analysis showed that EMT-high tumors are prone to be enriched with TAMs, revealing the association between the metabolic shift of aggressive tumors with pro-tumoral features of macrophages [108]. However, the “cause and consequence” relationship between the aggressiveness of tumor and macrophage phenotype is yet to be clarified, as TAMs can promote EMT via secreting TGFβ [109]. In addition to TGFβ as an EMT-inducer secreted by TAMs, co-culture of TAMs with HNSCC cells demonstrated that epidermal growth factor (EGF) from TAMs also triggers EMT via conveying its signal to activate extracellular signal-regulated protein kinase 1/2 (ERK1/2) [110]. Besides, tumor-associated dendritic cells (TADC) has been studied to render colon cancer cells to obtain EMT features and CSC properties by enhancing CD133 and aldehyde dehydrogenase (ALDH) via secreting C-X-C motif ligand 1 (CXCL1) [111]. These studies imply that the association of tumor aggressiveness and APC function is not a uni-directional event, but rather intertwined.

## Therapeutic approaches to restore the competent function of APCs

Based on recent observations on the dynamic interaction between tumor cells and TAMs through metabolites and nutrients within TME, an extensive amount of effort has been devoted to target metabolic features of TAMs to repolarize into M1 macrophages. TAMs expressing high levels of ARG1 render TME into an immunosuppressive environment by depleting arginine, which is critical for T cells and NK cells. Treatment of CB-1158, an ARG1 inhibitor under clinical trials, has been shown to restore the antitumor immunity exerted by T cells, both in vitro and in vivo syngeneic tumor model, and increase the population of pro-inflammatory macrophages [112]. Activation of PI3Kγ alters macrophages into immunosuppressive phenotype via activation of NF-κB-C/EBPβ. TAMs exhibited pro-inflammatory features upon the treatment of PI3Kγ blockade, which further led to enhanced antitumor immunity via altering T cell content [113]. Recently, the initiative role of protein kinase RNA-like ER kinase (PERK) promoting M2 polarization of TAM via upregulation of serine metabolism has been demonstrated. In this study, PERK inhibition with GSK2656157 not only delayed tumor progression, but also induced higher expansion of effector T cells [114]. Owing to its critical role in the M2 polarization of macrophages, targeting glutamine metabolism seems like a promising way to enhance antitumor immunity [115]. Inhibition of GS repolarized M2 macrophages into M1 macrophages, which further enhanced lymphocyte recruitment with less suppressive activity [66]. Furthermore, AMP-activated protein kinase (AMPK) activation by metformin treatment blocked M2 skewing of macrophages, which prevented metastasis of Lewis lung adenocarcinoma [116]. Targeting lactate signaling in TAMs can be another option to control tumor progression. G protein-coupled receptor 132 (GPR132) expressed on macrophages senses lactate within TME and leads macrophages to adopt M2-like phenotypes to promote breast cancer metastasis [117]. Treatment of rosiglitazone, an agonist of PPARγ, impeded tumor progression via inhibiting GPR132 expression on TAMs [118]. Furthermore, as the role of tryptophan metabolism has been demonstrated to be critical in modulating antitumor immunity, targeting tryptophan catabolism at different levels showed better antitumor immunity. Direct degradation of kynurenine with the administration of PEGlyated kynureninase substantially improved antitumor immunity against different types of cancers when combined with checkpoint inhibitors [89]. Furthermore, IDO-specific peptide (IDO vaccine) successfully reshaped immunosuppressive TME into immune-supportive TME, enriched with less M2 macrophages but with higher amount of M1 macrophages [119]. Nowadays, clinical trials targeting the tryptophan-kynurenine-AhR axis (e.g., AhR inhibitor) are under investigation in conjunction with checkpoint blockades [120]. Although studies on immunometabolism revealed targetable vulnerabilities which largely widened therapeutic options, targeting a single metabolic pathway of immune cells will lead to another round of metabolic rewiring of tumors as they retain high plasticity. Therefore, exploitation of metabolic targets that can improve antitumor immunity and perturb tumor metabolism simultaneously would be encouraged.

DCs (especially cDC1s) are central inducers of the immune response, which can further influence responsiveness to cancer therapies. Conventional cancer therapies, such as radiotherapy and chemotherapy, influence and require DC functions to improve therapeutic efficacy. Chemotherapy can trigger immunogenic tumor cell death, which results in releasing of stimulatory factors, such as apoptotic tumor cells, ATP and high-mobility-group box 1 (HMGB1). In turn, these factors play a role as alarmins that activate and mobilize DCs, which enhance the cross-presentation of tumor-associated antigens (TAAs) to elicit antitumor CD8 T cell responses [121]. Immunogenic chemotherapies, such as anthracycline- or oxaliplatin-treatment lead tumor cells to release ATP, which then recruits DCs and activates NOD-like receptor family, pyrin domain containing-3 protein (NLRP3) inflammasome, allowing the secretion of IL-1β [122]. In addition, there are several therapeutic approaches directly targeting DCs using DC-specific antibodies to deliver antigen/adjuvant or nanoparticles and adoptive transfer of autologous, antigen-loaded and activated DCs: DC activating factors, DC mobilizing agents, antigen-presenting, and antigen carriers [123]. The followings are the strategies targeting DCs to enhance antitumor immunity. (1) Providing exogenous activation signals, particularly derivatives of Agonists for TLRs or STING, can drive immunogenic DC activation. For example, treatment of tumor-bearing mice with NS-398 (COX-2 inhibitor), to reduce prostaglandin synthesis of tumor cells, can enhance the transcription factor *Zbtb46*, induce cDC lineage maturation, and enhance the activity of DCs [124–126]. Specifically, the lifespan of TLR-activated BMDCs is extended upon inhibition of mTOR due to reduced NO production as well as improved mitochondrial function [127]. (2) Vaccines and DC-specific antibodies to deliver antigen/adjuvant to increase antigen-specific T cell responses. DC vaccines include TAA-derived peptides, whole tumor lysates, and recombinant TAA-expressing viruses. It has been reported that DCs exhibit both accuracy and therapeutic efficacy after exposure to TAAs oxidized with mannan [123, 128, 129]. Furthermore, sarcosine-treated DC vaccines increase the migration ability of DCs, which is associated with the upregulation of COX1 and PIK3CG in B16F10 melanoma and glioma [130]. Dasatinib (tyrosine kinase inhibitor)-stimulated DC vaccines downregulate the IDO expression level and IDO-mediated tryptophan metabolism via inhibiting c-KIT [131]. Yellow fever vaccine (YF-17D) can enhance the activity of general control nonderepressible 2 kinase (GCN2), a sensor of amino acid starvation, thereby augmenting antigen presentation capacity of DCs, which leads to the modulation of an adaptive immune response [132]. Since vaccines have shown limited efficacy to date, combining adjuvants (GM-CSF or TLR Agonists) with vaccines are on the rise for in vivo provision [133]. For example, using a combination of OK432 (Picibanil), TLR7/8 ligand (CL097) and reduced PGE2 is manifested, as this combination stimulated the maturation of moDCs and increased the expression of co-stimulatory molecules and IL-12p70 production [134]. (3) Targeting metabolism in situ reprograms tumour-infiltrating DCs (TIDCs). For instance, inhibition of FAS using 5-(tetradecyloxy)-2-furoic acid (TOFA) or cerulenin prevents the accumulation of lipids, which in turn restores immunostimulatory activity and tumor control of TIDC [68, 135]. A rising number of preclinical studies regarding how to restore antitumor immunity by harnessing the metabolic properties of APCs will contribute to broaden the therapeutic options available to combat tumor (Table 2).

**Table 2:** Treatments targeting and modulating the metabolism of APCs to promote immunogenic function in cancers.

Compounds	Characteristics	Effect on APCs and immune consequences	Drug approved
CB-1158	ARG1 inhibitor	Increase the population of pro-inflammatory macrophages	Clinical trial
PI3Kγ antagonists	Activate of NF-κB-C/EBPβ	Enhance antitumor immunity via altering T cell content	Preclinical studies
GSK2656157	PERK inhibitor	Delayed tumor progression; Induce higher expansion of effector T cells	Preclinical studies
Methionine Sulfoximine	Glutamine synthetase inhibitor	Polarization of M2 macrophage into M2 macrophage	Preclinical studies
Rosiglitazone and anti-PD-1 mAb	PPARγ agonist	Inhibition of GPR132	Clinical trial
Metformin and anti-PD-1 mAb	Mitochondria Complex I inhibitor	Inhibition of M2 skewing	Clinical trial
PEGylated kynureninase	Kynurenine degradation	Reversal of immunosuppressive TME	Preclinical studies
Epacadostat And Pembrolizumab	IDO inhibitor		Clinical trial
IDO vaccine and Nivolumab	IDO-specific peptide		Clinical trial
BAY2416964 and Pembrolizumab	AhR inhibitor		Clinical trial
Anthracycline and Oxaliplatin	Chemotherapies	Active DC NLRP3 inflammasome; cDC2 infiltration	Approved
TLRs Agonists and STING	Ligands for PRRs	DC inflammatory cytokine secretion and co-stimulatory receptor upregulation	Numerous compounds in clinical trials
NS-398	COX-2 inhibitor	Reduce synthesis of tumor cell prostaglandin; Induce cDC lineage maturation and enhance activity	Preclinical studies
Mannan	Oxidize tumor antigens	DC has accurate and therapeutic efficacy	Preclinical studies
Dasatinib	Tyrosine kinase inhibitor	Downregulate expression and phosphorylate IDO; mediate tryptophan metabolism via inhibiting c-KIT	Clinical trial
OK432 (Picibanil), TLR7/8 ligand (CL097), reduced PGE2	GM-CSF, TLR agonists	Stimulate maturation of moDCs; increase IL-12p70 production	Imidazoquinoline Approved; OK432 approved
TLR-activated + Rapamycin	TLR agonist, mTOR inhibitor	Extend lifespan of DCs; improve mitochondria function	Preclinical studies
YF-17D	Yellow fever vaccine	Enhance GCN2 activation and autophagy to improve DC antigen presentation capacity	Active, not recruiting
TOFA or cerulenin	FAS inhibitors	Blockade of FA synthesis	Preclinical studies

Overview of factors and status of APC-based lines of research and clinical trials targeting metabolic features to boost the efficient immunotherapy. References are provided within the main text.

*CSF1R* colony-stimulating factor 1 receptor, *CXCR2* C-X-C chemokine receptor type 2, *Arg1* arginase 1, *PERK* protein kinase RNA-like ER kinase, *NLRP3* NLR family pyrin domain containing protein-3, *PRR* pattern recognition receptor, *GM-CSF* granulocyte-macrophage colony-stimulating factor, *IDO* indoleamine 2,3-dioxygenase, *TLR* toll-like receptor, *COX-2* cyclooxygenase 2, *GCN2* general control nonderepressible 2 kinase, *TOFA* 5-(tetradecyloxy)-2-furoic acid, *FAS* fatty acid synthesis.

Source:clinicaltrials.gov.

## Conclusion

Successful clearance of cancer largely relies on the proper activation of APCs. Although immunotherapy has revolutionized and fueled cancer therapies, a significant percentage of cancer patients do not benefit from cancer immunotherapies, partly due to low T cell infiltration or low tumor mutation burdens. Mounting evidence shows not only immunotherapy but also radiotherapy or chemotherapy requires the proper function of APCs to improve therapeutic efficacy. While tolerance and dysfunction of immune cells within TME are major hurdles that has to be overcome to fully harness the potential of APCs in cancer immunotherapy, immunostimulatory APCs are being recognized as a targetable source to elicit favorable adaptive immune responses. As expanded upon in previous sections, metabolic alteration of APCs is not a passive feature appearing during the immune response, but rather an active process that APCs engage to bolster adaptive immunity via regulating the functional status of T cells. Improved knowledge of how APCs are regulated in TME allowed therapeutic exploitation in clinical settings. However, direct targeting of metabolic pathways of APCs as a strategy to treat cancers has been marginally successful. Failure of targeting APC metabolism in clinical trials might be due to several reasons: insufficiency of targeting a single metabolic pathway to overcome the metabolic thresholds of APCs within TME, which is more harsh than preclinical models, and plasticity of tumor cells that can further rewire their metabolism. Therefore, targeting metabolism in combination with other therapies, including checkpoint inhibitors, will be a promising avenue. Moreover, investigating further on how multiple metabolic pathways in APCs are intertwined to interpret different metabolic signals within TME will help figure out a better target and refine the strategies to target the metabolism of APCs for reverting the dysfunctionality of APCs. A better understanding of the metabolic features of APCs in TME will change the landscape of cancer therapies that can improve the survival of patients with better outcomes.

## Data Availability

This manuscript does not contain original experimental data.
